# Immunosenescence shapes the tumor immune microenvironment and limits PD-1/PD-L1 blockade efficacy in older patients with cancer

**DOI:** 10.3389/fimmu.2026.1781004

**Published:** 2026-07-21

**Authors:** Xiaoyu Niu, Ziwaregul Nur, Wenkang Xie, Yang Sun, Longhao Wang, Yuanyuan Zheng

**Affiliations:** 1 Department of Oncology, The First Affiliated Hospital of Zhengzhou University, Zhengzhou, Henan, China; 2 Internet Medical and System Applications of National Engineering Laboratory, the First Affiliated Hospital of Zhengzhou University, Zhengzhou, Henan, China; 3 Department of Oncology, Henan Provincial People’s Hospital, Zhengzhou, Henan, China; 4 Department of Neurosurgery, Henan Provincial People's Hospital, Zhengzhou, Henan, China; 5 Henan Health Commission Key Laboratory of Cancer Radiation Injury and Protection, The First Affiliated Hospital of Zhengzhou University, Zhengzhou, Henan, China; 6 The First Clinical School of Medicine, Zhengzhou University, Zhengzhou, Henan, China

**Keywords:** gut microbiome, immunosenescence, inflammaging, metabolic reprogramming, myeloid-derived suppressor cells, PD-1/PD-L1 blockade

## Abstract

As the global population ages, immunosenescence is emerging as a critical determinant of cancer outcomes in older adults. Although programmed cell death protein 1/protein programmed death-ligand 1 (PD-1/PD-L1) blockade has significantly improved the treatment of multiple malignancies, its efficacy in older patients is highly heterogeneous, and the biological basis for this variability remains incompletely understood. Current evidence indicates that immunosenescence reshapes antitumour immunity through thymic involution, reduced T cell receptor diversity, chronic low-grade inflammation, and expansion of immunosuppressive cell populations, thereby impairing antigen presentation, weakening T cell activation and effector function, promoting terminal T cell exhaustion, and reinforcing suppressive tumour microenvironments. Together, these changes form an important mechanistic basis for the limited benefit of PD-1/PD-L1 blockade in older patients. Meanwhile, potentially targetable processes, including metabolic dysregulation, mitochondrial dysfunction, defective autophagy–mitophagy, redox imbalance, and gut microbiota dysbiosis, are increasingly recognized as modifiable contributors to age-associated resistance to immunotherapy. In this Review, we discuss how immunosenescence remodels antitumour immunity and constrains responses to PD-1/PD-L1 blockade in older patients, and we summarize potential strategies to improve immunotherapeutic efficacy in this population. These insights may inform future mechanistic studies, biomarker discovery, and the development of age-adapted therapeutic strategies.

## Introduction

Aging is an inevitable biological process characterized by a systematic decline in the regenerative capacity of cells, manifested as deterioration in structure and function across various tissues. It profoundly alters the immune environment and has a significant impact on the development and treatment of cancer (1). In parallel with global population aging, the incidence of malignancies continues to rise, particularly among the elderly, who represent the fastest-growing demographic of cancer patients. Age-related comorbidities in elderly populations are linked to immunosenescence, a term coined by Dr. Roy Walford to describe the progressive erosion of immune competence with advancing age (2). Immunosenescence is further defined by hallmark features such as contracted T-cell receptor (TCR) diversity, expanded memory/effector T-cell pools, thymic atrophy, naïve T-cell depletion, prolonged inflammatory signaling, and increased expression of immune inhibitory receptors (3). Simultaneously, aging promotes a state of chronic, low-grade inflammation (inflammaging), marked by elevated levels of pro-inflammatory cytokines such as IL-6, TNF-α, and IL-1β, and oxidative stress via reactive oxygen species (ROS) accumulation, and heightened autoimmune susceptibility (4). These age-driven immune perturbations likely diminish antitumor immunity and responsiveness to immunotherapy. Immunosenescence may compromise tumor immunosurveillance, thereby elevating cancer risk. This is corroborated by the elevated incidence of virally induced malignancies in immunocompromised populations, which underscores the immune system’s critical role in suppressing oncogenesis (5). Consequently, age-related immune dysfunction may amplify tumorigenesis driven by genetic or epigenetic dysregulation of oncogenes or tumor suppressors.

Notably, the emergence of immune checkpoint inhibitors (ICIs) has redefined cancer therapeutics. PD-1/PD-L1 blockade relieves inhibitory signaling between PD-L1-expressing tumor or myeloid cells and PD-1^+^ T cells, thereby restoring antitumor T cell activity; however, this effect may be limited in aged hosts because antigen presentation and functional T cell pools are impaired (6, 7). However, most pivotal clinical trials have underrepresented older individuals, leading to a limited understanding of how aging affects immunotherapy efficacy. Age-related immune dysfunction—often characterized by immunosenescence, chronic inflammation (inflammaging), and altered immune cell composition—may attenuate antitumor immunity and impact therapeutic outcomes (8). Consistent with these findings, preclinical murine models have demonstrated age-associated impairments in CD8^+^ T cell activation and tumor eradication following ICI therapy (9). Given that the therapeutic efficacy of ICIs hinges upon the reinvigoration of antitumor immunity, age-related immunosenescence—characterized by a contraction of T cell receptor (TCR) diversity, chronic low-grade inflammation, and aberrant cytokine signaling—likely dictates both the efficacy and toxicity profiles of ICIs in older populations (10).

Furthermore, the aging tumor microenvironment (TME) undergoes extensive immunological and structural remodeling that compromises key antitumor immune functions, including immune cell infiltration, antigen presentation, and cytokine signaling (11). Accumulating evidence indicates that aged TMEs are enriched with immunosuppressive components, such as myeloid-derived suppressor cells (MDSCs), regulatory T cells (Tregs), and senescent stromal cells, while exhibiting a notable reduction in cytotoxic T lymphocyte (CTL) infiltration (12). These alterations can dampen the efficacy of ICIs by creating physical and biochemical barriers to immune cell trafficking and activation. Given these complexities, elucidating the interplay between aging and immunotherapy response is essential for optimizing treatment strategies in older cancer patients. Addressing this gap will enable the development of predictive biomarkers, age-adjusted clinical trial designs, and tailored immunotherapeutic strategies to improve outcomes in aging populations.

Based on the above background, this article will systematically review the remodeling effect of aging on the anti-tumor immune response, focusing on how these age-related changes shape the intrinsic tolerance basis of elderly patients to PD-1/PD-L1 blocking therapy, and summarizing the potential intervention pathways that may improve the immune treatment response.

## Clinical evidence for PD-1/PD-L1 blockade in older patients

Available clinical evidence indicates that older patients can still benefit from PD-1/PD-L1 blockade, but therapeutic outcomes are heterogeneous. A meta-analysis and meta-regression of 30 randomized phase II/III trials involving 17,476 patients with advanced solid tumors showed comparable survival benefits from ICIs in patients aged ≥65 years and younger patients, suggesting that chronological age alone should not preclude immunotherapy (13). However, evidence in very old patients is less consistent. A study-level meta-analysis focused on patients aged ≥75 years reported attenuated survival benefit from anti-PD-1/PD-L1 therapy in this population, except in melanoma, indicating that biological immune aging may be more informative than chronological age alone (14).

Tumor-specific evidence further supports this heterogeneity. In a pooled analysis of KEYNOTE-010, KEYNOTE-024, and KEYNOTE-042, pembrolizumab improved overall survival and showed a favorable safety profile compared with chemotherapy in patients aged ≥75 years with PD-L1-positive advanced NSCLC, particularly in those with PD-L1 tumor proportion score ≥50% (15). More recently, a systematic review and meta-analysis of elderly patients with advanced NSCLC confirmed that ICIs improved overall survival and progression-free survival compared with chemotherapy, whereas age ≥75 years, ECOG performance status ≥2, and PD-L1 expression <1% appeared to negatively affect efficacy (16). These findings suggest that anti-PD-1/PD-L1 therapy remains clinically relevant for selected older patients, but its benefit is shaped by tumor type, PD-L1 expression, performance status, frailty, comorbidities, and the extent of immunosenescence.

## Age-related changes in the immune system

### Effective immune responses are formed in the tumor microenvironment

The interplay between immune responses and tumorigenesis is fundamentally paradoxical. Under physiological conditions, immune cells are recruited to neoplastic sites to exert cytotoxic effects and preserve tissue homeostasis. Yet, tumors and their stromal counterparts actively subvert immune surveillance by suppressing CD8^+^ T cell cytotoxicity and fostering CD4^+^ T cell tolerance, thereby driving angiogenesis, metastatic dissemination, and apoptotic resistance (17, 18). Tumor progression follows a triphasic immunoediting paradigm—elimination, equilibrium, and escape—wherein bidirectional interactions between malignant and immune cells shape disease evolution (19). Genetic instability and immunoselection pressure enable tumors to evade immune detection via antigen loss or immunomodulatory adaptation.

Critically, the prognostic value of intratumoral immunity is underscored by the spatial density, functional quality, and clonal diversity of tumor-infiltrating lymphocytes (TILs). A favorable prognosis correlates with robust infiltration of CD8^+^ cytotoxic T lymphocytes (CTLs), CD45RO^+^ memory T cells, and pro-inflammatory cytokines (e.g., IFN-γ, TNF-α) within the TME (20, 21). Despite the immune system’s role as a sentinel of antitumor immunity, tumors evolve sophisticated escape mechanisms. The TME orchestrates immune evasion through immunosuppressive factor secretion (e.g., TGF-β, IL-10) and recruitment of regulatory immune subsets (e.g., Tregs, MDSCs), which collectively foster tumor progression (22, 23). Furthermore, physicochemical barriers within the TME—including hypoxia, dysregulated extracellular matrix (ECM) remodeling, and nutrient competition—restrict immune cell infiltration and effector function (24). In addition, stromal and myeloid components such as cancer-associated fibroblasts (CAFs) and tumor-associated macrophages (TAMs) further reinforce immune exclusion in the TME. CAFs can suppress CD8^+^ T cell infiltration and confer resistance to immune checkpoint blockade, while TAMs can physically restrict CD8^+^ T cell access to tumor cells and limit the efficacy of anti-PD-1 therapy (25, 26). These findings suggest that effective PD-1/PD-L1 blockade requires not only T cell reinvigoration but also remodeling of the stromal–myeloid suppressive niche.

Emerging therapeutic paradigms seek to reprogram the TME by dual targeting of immunosuppressive pathways (e.g., PD-1, CTLA-4) and potentiation of immune-activating signals. Notably, ICIs and adoptive cell therapies (e.g., CAR-T cells) exemplify this strategy, leveraging engineered immune cells or receptor blockade to counteract tumor-mediated suppression (27). Thus, the TME emerges as a linchpin of therapeutic efficacy, and its modular reprogramming presents significant potential for improving oncologic outcomes.

### Reduced T cell output, atrophy of the thymus gland, and immunodeficiency of adaptive immunity

**Figure 1:**
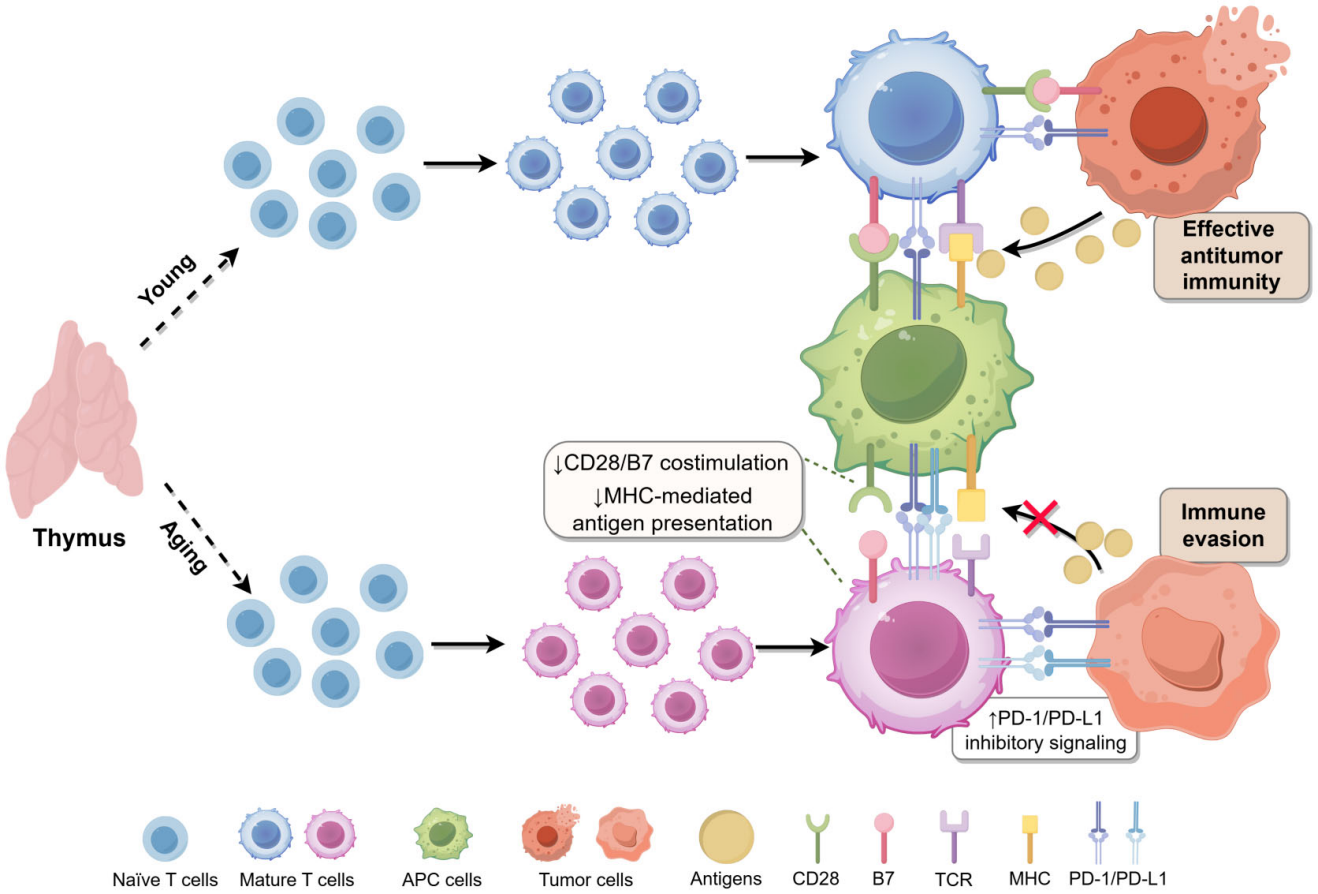
Aging-associated thymic involution and impaired T cell priming promote immune evasion. In young hosts, preserved thymic output supports the generation of naïve and functionally competent T cells, while effective antigen presentation and CD28/B7 costimulation promote antitumor immunity. In aged hosts, thymic involution reduces naïve T cell output and functional fitness, accompanied by impaired APC-mediated antigen presentation, weakened costimulatory signaling, and enhanced PD-1/PD-L1 inhibitory signaling. Together, these changes diminish T cell activation and effector responses, facilitating tumor immune evasion.

The canonical model of tumor immunoediting encompasses three sequential phases: elimination, equilibrium, and escape. Notably, age-driven immune attrition can subvert this trajectory; by crippling early immunosurveillance networks, immunosenescence enables nascent malignancies to circumvent the elimination phase, thereby precipitating a rapid transition directly into equilibrium or immune evasion (**Figure 1**) (1). Deciphering how aging remodels the immune microenvironment is pivotal for predicting immunotherapy outcomes. Elderly patients exhibit distinct tumor responses compared to younger cohorts, driven largely by age-related declines in T cell output and function (28). T cells, the primary mediators of antitumor immunity, experience profound numerical and functional deficits in both CD4^+^ and CD8^+^ subsets during immunosenescence. Thymic involution, a hallmark of aging, exacerbates this decline by reducing naïve T cell diversity, impairing effector T cell proliferation, and promoting memory T cell accumulation due to chronic antigen exposure, alongside expansion of immunosuppressive Tregs (29–31). Chronic thymic atrophy further compromises T cell resilience, rendering these cells susceptible to exhaustion.

### Immune checkpoint activation, chronic antigen stimulation and T cell exhaustion

During tumor evolution, accumulating mutations generate neoantigens that distinguish cancer cells from normal cells. Paradoxically, tumors exploit immune checkpoint pathways (e.g., PD-L1) to suppress TILs and activate immunosuppressive Tregs, thereby evading immune destruction (32, 33). Pathologically expressed inhibitory ligands (e.g., within the TME) suppress TIL activation while promoting Treg expansion—a dual mechanism exploited by tumors to evade immune surveillance. Conversely, blockade of these ligands can restore endogenous antitumor immunity (34). To prevent immunopathology during foreign antigen recognition, T cells rapidly engage immune checkpoint pathways. A prototypical example is the PD-1/PD-L1 axis, where PD-L1-expressing tumors ligate PD-1 on T cells to terminate effector responses (35).

Chronic antigen exposure—as seen in persistent infections or the TME—drives T cell exhaustion, a hallmark of immunosenescence. This dysfunctional state is defined by reduced proliferative capacity, diminished effector function, and upregulated coinhibitory receptors (PD-1, TIM-3, CTLA-4, BTLA, CD160, LAG-3, 2B4), coupled with reduced functional avidity for antigens (35, 36). These molecules serve as valuable probes to dissect the transcriptional programs underpinning immunosenescence in cancer patients and offer actionable targets for reversing age-related immune dysfunction. Recent evidence suggests that T cell exhaustion in aging is not merely a transient functional state but is reinforced by a stable epigenetic landscape. The high expression of the transcription factor TOX leads to irreversible chromatin remodeling, essentially ‘locking’ the cells in a dysfunctional state where effector genes (e.g., IFN-γ, GZMB) are physically inaccessible (37). This epigenetic rigidity suggests that in elderly patients, PD-1/PD-L1 blockade may face a biological ceiling unless paired with strategies that target the epigenetic machinery, such as HDAC inhibitors or DNA methyltransferase inhibitors (38).

### Insufficient support for antigen presentation and T cell activation

Aging further disrupts adaptive immunity through multiple mechanisms: (1) thymic loss of MHC antigens critical for self-tolerance and antigen presentation (39); (2) dysregulation of TCR signaling fidelity (40); and (3) proinflammatory cytokine-driven oxidative stress, wherein elevated interleukin IL-6, IL-1β, or TNF-α induce ROS overproduction in CD4^+^ T cells, impairing effector function via inhibition of key signaling cascades (41). The above changes indicate that the overall ability of elderly hosts to process antigens and activate T cells has declined, which lays the foundation for the limited efficacy of subsequent ICI treatments.

## Intrinsic barriers to PD-1 blockade therapy related to aging

### Activation of the immunosuppressive network under the aging phenotype

**Figure 2:**
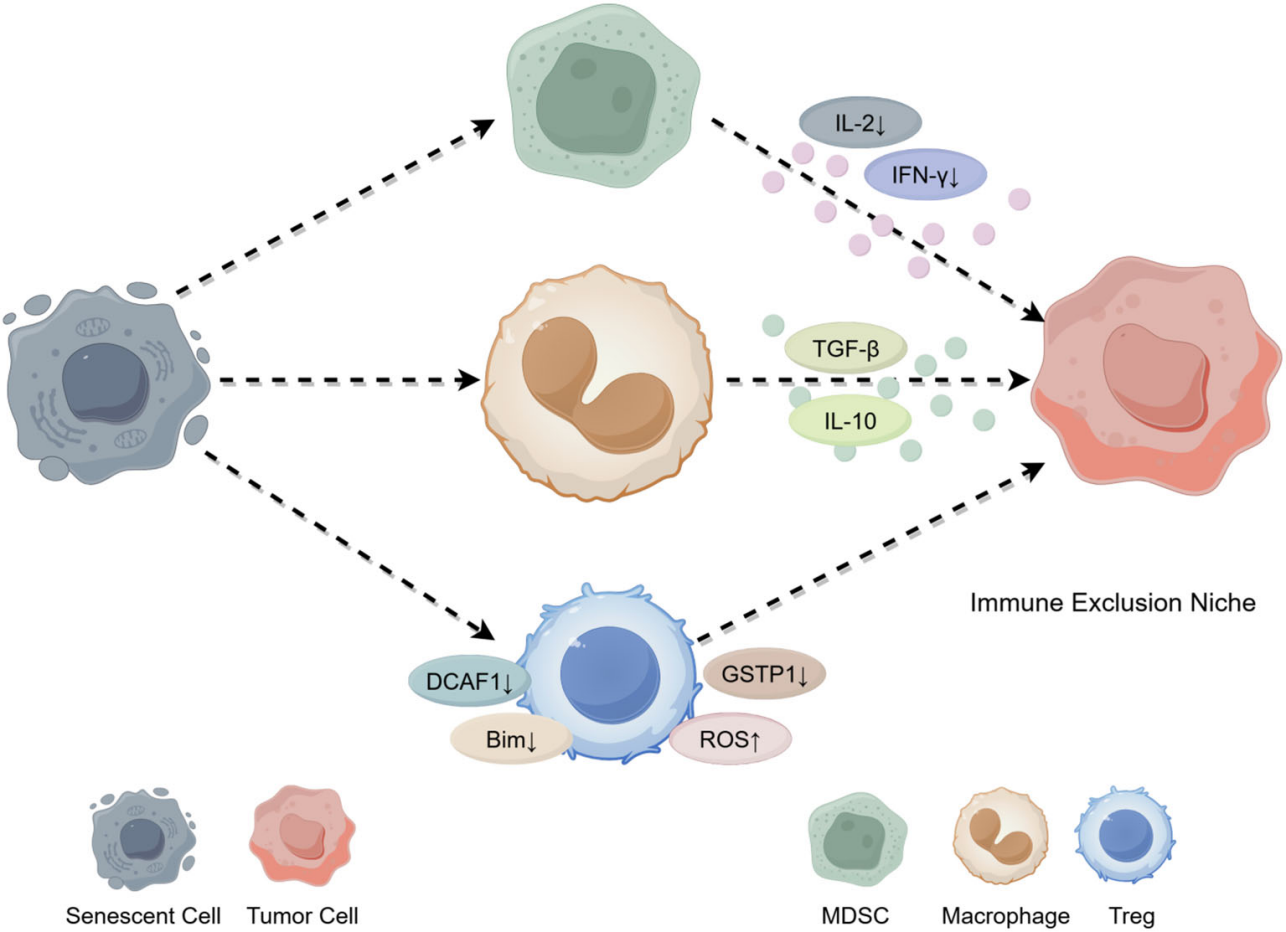
Senescent cells reinforce an immunosuppressive microenvironment through MDSCs, macrophages, and Tregs. Senescent cells promote the expansion and activation of multiple suppressive cell populations in the aged tumor microenvironment. MDSCs dampen antitumor immunity by suppressing T cell-associated cytokine responses, including IL-2 and IFN-γ. Tumor-associated macrophages enhance protumor inflammation and immune suppression through factors such as TGF-β. In parallel, aged Tregs acquire enhanced suppressive activity through dysregulation of the DCAF1/GSTP1/ROS axis and reduced Bim expression. Together, these suppressive populations form localized immunosuppressive niches that favor immune exclusion and tumor progression.

Aging is characterized by a systemic increase in proinflammatory responses across immune and nonimmune tissues, driven by cumulative endogenous and environmental damage. Inflammaging, represents a hallmark of aging and is mechanistically linked to immunosuppressive network activation, diminished cancer immunity, and age-related immune decline (**Figure 2**) (42).

### Expansion of MDSCs and their immunosuppressive effects

MDSCs are heterogeneous immunosuppressive myeloid populations that expand during chronic inflammation, cancer, and aging (43). In older adults and elderly cancer patients, increased circulating CD33^+^HLA-DR-MDSCs have been reported, suggesting that aging-associated myeloid skewing may reinforce systemic and tumor-localized immune suppression (44). Within the aged TME, MDSCs can suppress T cell activation through arginase-1, inducible nitric oxide synthase, ROS, IL-10, and TGF-β, and may cooperate with other myeloid populations to form spatially restricted suppressive niches associated with exhausted CD8^+^ T cells (45). These findings provide a mechanistic link between inflammaging, myeloid-driven immune exclusion, and reduced responsiveness to PD-1/PD-L1 blockade.

### Abnormal polarization of macrophages and enhanced pro-tumor inflammation

TAMs, derived circulating from monocytes recruited to inflammatory niches, orchestrate a broad spectrum of protumorigenic programs. Recapitulating the behavior of other immunosuppressive myeloid subsets, TAMs actively catalyze malignant proliferation stimulate neovascularization, facilitate metastatic spread, and profoundly subvert adaptive antitumor immunity. These cells act as central orchestrators of cancer-associated inflammation, a tumor-promoting inflammatory network driven by malignant cells, stromal cells, and infiltrating immune cells that supports angiogenesis, matrix remodeling, immune suppression, and therapeutic resistance (46, 47). Immunosenescence exacerbates immune cell dysfunction, with aging macrophages exhibiting impaired pathogen recognition and clearance. Jackaman et al. demonstrated that aged mice exhibit increased accumulation of immunosuppressive M2 macrophages in the spleen, lymph nodes, and bone marrow, which secrete elevated TGF-β levels and suppress antitumor responses (48). Notably, macrophage depletion in aged murine models enhances antitumor T cell activity and improves IL-2/anti-CD40 immunotherapy efficacy, achieving up to 78% tumor regression (49). Zhou et al. further revealed that IL-4-STAT6 signaling activation restores macrophage phagocytic capacity by upregulating DNA repair genes (e.g., via homologous recombination and Fanconi anemia pathways), ameliorating age-related immune deficits and extending healthy lifespan in aged mice (50). Tumor-associated macrophages occupy distinct spatial niches rather than being randomly distributed, and age-related myeloid remodeling may further strengthen macrophage-mediated restriction of T cell infiltration and function (51). These findings suggest that age-skewed macrophage polarization amplifies cancer-associated inflammation and may promote tumor progression, immune escape, and resistance to immunotherapy.

### The accumulation of Tregs and their inhibitory effect on anti-tumor immunity

Aging is associated with profound phenotypic and functional alterations in Tregs. Age-dependent increases in Treg frequency occur systemically, including in the blood, spleen, and peripheral lymph nodes (52). In aged murine models, Treg accumulation directly suppresses antitumor immunity, mechanistically linking Treg dysregulation to cancer progression (53). Tregs drive immunosuppression across multiple cancer types, correlating with poor clinical outcomes, while age-related imbalances in Treg homeostasis exacerbate immune dysfunction (54). Mechanistically, aged Tregs exhibit reduced expression of the pro-apoptotic factor Bim, enhancing their survival and promoting pathological accumulation (55). Notably, gut microbiota transplantation from aged to young germ-free mice recapitulates age-associated immune phenotypes, including splenic T cell hyperactivation, Treg expansion, and elevated gut inflammation, implicating age-related dysbiosis in Treg-driven inflammation (56). The molecular basis of Treg senescence involves dysregulation of the DCAF1/GSTP1/ROS axis. In inflammatory bowel disease models, aged Tregs exhibit downregulated DDB1- and CUL4-associated factor 1 (DCAF1), while restoring glutathione-S-transferase P (GSTP1) activity or scavenging ROS rejuvenates Treg proliferative and functional capacity (57). Recent spatial analyses identified a peri-lymphatic Treg–mregDC niche that suppresses antigen trafficking and limits antitumor T cell priming, a localized suppressive architecture that may be particularly relevant in aged tumors (58). Importantly, PD-1/PD-L1 blockade may also influence Tregs rather than acting exclusively on exhausted effector T cells. PD-1 blockade can expand intratumoral Tregs and thereby limit immunotherapeutic efficacy, suggesting that in Treg-enriched aged TMEs, the therapeutic outcome may depend on the balance between effector T cell reinvigoration and Treg-mediated suppression (59, 60). Thus, while Tregs are essential for maintaining immune homeostasis during aging, their excessive immunosuppression contributes to immunosenescence, elevating susceptibility to age-associated pathologies such as cancer and chronic infections. Targeted modulation of Treg function—particularly through the DCAF1/GSTP1/ROS axis—represents a promising therapeutic avenue to counteract immune aging and enhance antitumor/anti-pathogen immunity.

### Insufficient antigen presentation and CD8^+^ T cell support limit the ICI response

Immune checkpoint inhibitors have demonstrated effectiveness in the treatment of implantable tumors in young mice, but have shown limited efficacy in older mice, which correlates with a reduced frequency of CD8^+^ T cells in the elderly (7). Moreover, hyperactive dendritic cells (DCs) in young mice play a crucial role in inducing antitumor immunity by enhancing the activity of CD8^+^ T cells (61). Interestingly, similar defects in T cell populations have also been observed in elderly humans. A recently published study found that inhibition of the matrix-binding receptor SDC1 in tumor cells resulted in increased accumulation of CD8^+^ T cells, thereby enhancing the immune response to anti-PD-1 treatment (62). Therefore, therapeutic strategies aimed at overcoming the current limitations in the immune microenvironment and enhancing CD8^+^ T cell activation within the TME could significantly improve the efficacy of PD-1 therapies, potentially benefiting a broader patient population.

### Aging-associated determinants of limited responsiveness to PD-1/PD-L1 blockade

Aging-associated T cell dysfunction is a major determinant of limited response to PD-1/PD-L1 blockade. Senescent tumor cells may further reinforce resistance by upregulating senescence-associated secretory phenotype (SASP)-associated programs and displaying heterogeneous PD-L1 expression (63). In parallel, increased PD-1 expression, impaired survival, and reduced effector fitness of aged CD8^+^ T cells restrict the reinvigoration potential of ICIs (64–66). Clinical observations in NSCLC likewise show that immunosenescent T cell phenotypes are associated with inferior response and survival outcomes after PD-1/PD-L1 inhibition (67).

In addition to T cell-intrinsic dysfunction, aging may also impair PD-1/PD-L1 blockade through biophysical barriers within the TME (68). Hypoxia, dysregulated ECM remodeling, and other physicochemical constraints can limit immune-cell trafficking and effector function (27). In aged tumors, these structural changes may further reduce CD8^+^ T cell access to tumor nests and reinforce immune exclusion, thereby linking stromal remodeling to clinical resistance to immunotherapy (68).

Immune aging also affects the toxicity profile of ICIs. Age-related systemic inflammation has been linked to increased off-target toxicity during anti-PD-1 therapy, and higher rates of immune-related adverse events may further compromise treatment continuity and benefit in older patients (69, 70).

### Immune aging simultaneously affects the efficacy and toxicity of ICI

Notably, age-related systemic inflammation exacerbates ICI toxicity. A recent study reported that age-associated CD4^+^ T cell-derived CXCL13 overexpression drives off-target organ toxicity during anti-PD-1 therapy (63). Elderly patients also experience higher rates of immune-related adverse events (irAEs; 33% vs. 25%, P = 0.03), with irAE-driven treatment discontinuation contributing to reduced ICI efficacy (64). These data position immunosenescence as a dual determinant of ICI safety and efficacy. Given the age-dependent accumulation of PD-L1+ senescent cells and systemic inflammaging, holistic therapeutic strategies—such as senolytic agents to eliminate senescence-associated immunosuppression—may synergize with ICIs to improve outcomes in elderly patients.

### Abnormal key signaling pathways and the amplification of the aging immunosuppressive microenvironment

The core functional alterations of major immune cell subsets during immunosenescence and their effects on antitumor immunity and tumor progression are summarized in **Table 1**.

**Table 1:** Core functional alterations of immune cell subsets during immunosenescence and their impact on antitumor immunity and tumor progression.

Immune cell subset	Core alterations during immunosenescence	Impact on antitumor immunity and tumor progression	Representative references
CD8^+^ T Cells	Decreased TCR affinity, reduced release of perforin/granzyme, and weakened cytotoxic activity; Increased expression of PD-1/TIM-3/LAG-3, prone to an exhausted state; Proliferation arrest and abnormal mitochondrial function (decreased ATP, increased ROS)	Reduced accuracy in tumor antigen recognition, leading to immune evasion; Weakened cytotoxicity, resulting in decreased tumor cell apoptosis rate; Reduced responsiveness to PD-1/PD-L1 blockade; excessive ROS and mitochondrial dysfunction may further impair effector function and favor tumor adaptation	(9, 10, 27, 29, 30, 55)
CD4^+^ T Cells	Imbalanced function of Th1/Th2/Th17 (decreased IFN-γ/IL-17, increased IL-10); Enhanced immunosuppression by Treg (increased secretion of TGF-β/IL-10); Decreased expression of co-stimulatory molecule CD28, weakened auxiliary activation ability	Diminished cellular immune response, insufficient activation of CTLs/B cells; Strengthened immunosuppressive microenvironment, accelerating tumor evasion; Disordered humoral immunity, reduced production of specific antibodies	(28, 55, 58)
Regulatory T Cells (Treg Cells)	Increased secretion of immunosuppressive factors (TGF-β/IL-10); Increased surface expression of CTLA-4, competitively binding to CD80/CD86 on DC cells; Expanded range of inhibition on effector T cells and NK cells; Dysregulation of the DCAF1/GSTP1/ROS axis may further enhance suppressive activity in aged Tregs	Significantly weakened anti-tumor activity of CTLs/NK cells due to a reinforced immunosuppressive microenvironment; Blocks T cell activation by DC cells, hindering adaptive immune response; Accelerates immune evasion of tumor cells, promoting tumor proliferation and metastasis	(48–54, 69)
Memory T Cells	Decreased self-renewal ability, reduced size of memory cell pool; Decreased migration ability, weakened speed/strength of antigen response; Insufficient ability to recognize mutant tumor antigens	Slow initiation of response during tumor recurrence, failing to rapidly mobilize effector cells; Insufficient memory reserve, difficulty in coping with tumor re-invasion; Easy evasion of surveillance after tumor antigen mutation	(4, 20–23, 27)
Macrophages	Polarization shift toward M2 phenotype, decreased phagocytic/antigen-presenting ability; Increased production of pro-angiogenic factors (VEGF/MMPs) and anti-inflammatory factors (IL-10)	Weakened anti-tumor cytotoxicity, failing to eliminate tumor cells; Promotes tumor angiogenesis, providing nutrients for proliferation; Helps establish immunosuppressive niches that facilitate immune exclusion and tumor evasion	(39, 43–47)
Natural Killer Cells (NK Cells)	Decreased expression of activating receptors (NKG2D), increased expression of inhibitory receptors (KIR); Reduced release of cytotoxic granules and decreased secretion of IFN-γ; Decreased migration ability, difficulty in infiltrating tumor tissues	Insufficient clearance of early malignant cells, increased risk of tumorigenesis; Weakened direct cytotoxicity, accelerating tumor proliferation and metastasis; Fails to activate adaptive immunity, resulting in a weak anti-tumor effect	(13, 16)
Dendritic Cells (DC Cells)	Maturation disorder, decreased expression of MHC/co-stimulatory molecules (CD80/CD86); Decreased antigen-presenting ability and IL-12 secretion; Decreased ability to migrate to lymph nodes	Fails to effectively present tumor antigens and prime T cells, thereby limiting initiation of adaptive immunity; Insufficient activation of CTLs, leading to tumor immune evasion; Absent anti-tumor immune response, accelerating tumor progression	(31, 55)
B Lymphocytes	Decreased proliferation and differentiation, reduced antibody production and weakened affinity; Decreased memory B cells, delayed secondary immune response; Possible production of autoantibodies, inducing chronic inflammation	Decreased proliferation and differentiation, reduced antibody production and weakened affinity; Decreased memory B cells, delayed secondary immune response; Possible production of autoantibodies, inducing chronic inflammation Insufficient anti-tumor antibodies, failing to mark tumor cells for clearance; Weakened humoral immunity, reduced cytotoxic efficiency of NK cells/macrophages; Chronic inflammation may promote formation of the tumor microenvironment	(17)
Neutrophils	Decreased phagocytic and cytotoxic ability, abnormal release of NETs; Decreased pro-inflammatory factors, increased anti-inflammatory/pro-tumor factors; Delayed apoptosis, prone to abnormal accumulation	Weakened clearance of early tumor cells, failing to inhibit progression; Abnormal NETs promote tumor adhesion and migration; Chronic inflammation provides favorable conditions for tumor proliferation	(16, 44)

TCR, T cell receptor; PD-1, programmed cell death protein 1; TIM-3, T cell immunoglobulin and mucin-domain containing-3; LAG-3, lymphocyte activation gene-3; ROS, reactive oxygen species; IFN-γ, interferon gamma; IL, interleukin; TGF-β, transforming growth factor beta; CD, cluster of differentiation; Treg, regulatory T cell; CTL, cytotoxic T lymphocyte; DC, dendritic cell; NK, natural killer cell; VEGF, vascular endothelial growth factor; MMP, matrix metalloproteinase; NET, neutrophil extracellular trap.

The Ras/Raf/MEK/ERK and PI3K/PTEN/Akt/mTOR signaling pathways critically regulate cell growth, metabolism, ribosome biogenesis, and senescence, while also driving DNA damage responses and aging-associated phenotypes (65–67). Hyperactivation of these pathways correlates with poor cancer prognosis, premature aging, and heterogeneous patient responses to targeted therapies (66). Pharmacological inhibition of MEK, PI3K, or mTOR suppresses malignant cell proliferation and mitigates aging-related loss of proliferative potential, suggesting that targeted modulation of these pathways may counteract age-associated cellular dysfunction (69–71).

**Figure 3:**
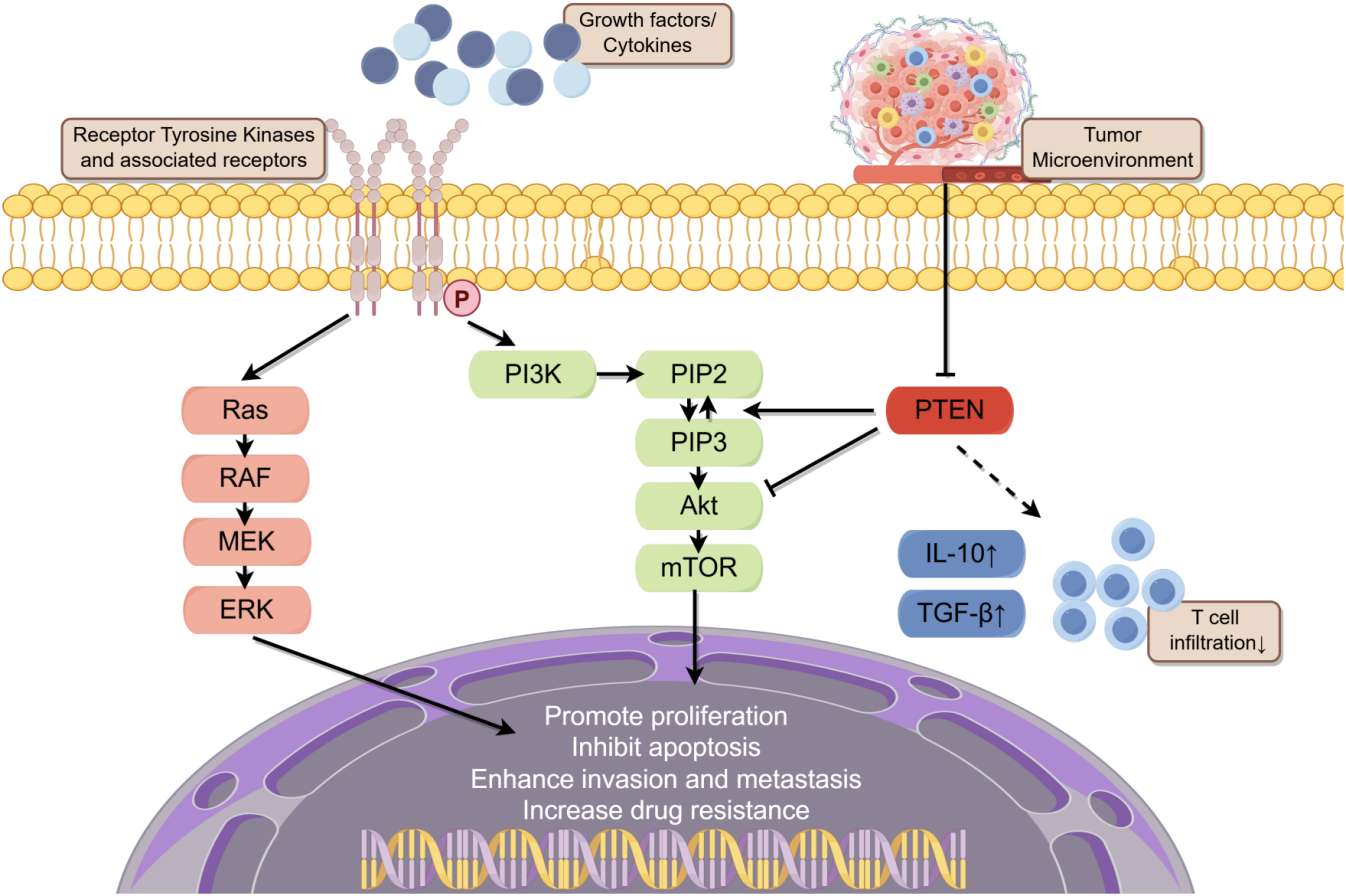
Aging-associated oncogenic and immunosuppressive signaling pathways amplify resistance to PD-1/PD-L1 blockade. The Ras/RAF/MEK/ERK and PI3K/PTEN/AKT/mTOR pathways regulate cell growth, metabolism, senescence, and survival. Hyperactivation of these pathways promotes tumor progression and contributes to aging-associated dysfunction. In particular, PI3K/AKT/mTOR activation and PTEN loss enhance immunosuppressive signaling, increase IL-10 and TGF-β, reduce T cell infiltration, and thereby support resistance to PD-1/PD-L1 blockade. Targeted inhibition of these pathways may help reprogram the aged tumor microenvironment and improve immunotherapeutic responsiveness.

Notably, PI3K-Akt-mTOR signaling promotes tumorigenesis through multifaceted mechanisms, including PTEN suppression—a tumor suppressor that regulates cell survival, migration, and metabolism. PTEN loss reduces T cell infiltration, elevates immunosuppressive cytokines (e.g., IL-10, TGF-β), and confers resistance to PD-1/PD-L1 blockade, as demonstrated in preclinical models (72). Selective PI3K-Akt inhibitors synergize with ICIs to restore antitumor immunity in ICI-resistant tumors (73). Aging reshapes the TME through ECM remodeling, enhancing tumor invasiveness and amplifying the SASP. Senescent cells within the aged TME exacerbate chronic inflammation and recruit Tregs via PD-1/PD-L1 axis activation, which paradoxically enhances Treg proliferative capacity and immunosuppressive function (**Figure 3**; 59). Collectively, these findings implicate aging as a key modulator of TME immunosuppression, potentially influencing ICI efficacy through Treg dynamics The principal aging-associated oncogenic and immunosuppressive signaling pathways that contribute to resistance to PD-1/PD-L1 blockade are summarized in **Figure 3**.

## Potential strategies to improve immunotherapy outcomes in older patients

### Targeted metabolism and mitochondrial dysfunction

Cellular senescence is characterized by profound metabolic reprogramming, including enhanced glycolysis, dysregulated mitochondrial metabolism, and altered autophagic flux. Activated oncogenes and genotoxic therapies induce therapy-induced senescence (TIS), a state marked by elevated glucose utilization and ATP overproduction in murine lymphoma models (74). Senescent cells counteract proteotoxic stress by upregulating endoplasmic reticulum (ER) stress, activating the unfolded protein response (UPR), and amplifying ubiquitination pathways, which collectively drive the SASP (74). A central objective in aging research is the development of therapeutics targeting shared pathways underlying age-related pathologies. Polyphenolic compounds, such as resveratrol, exhibit pleiotropic benefits by mitigating oxidative stress, suppressing inflammation, and modulating apoptosis through mitochondrial reprogramming (75). These findings position resveratrol as a promising prototype for therapies targeting metabolic vulnerabilities in aging and cancer.

### Targeted autophagy - mitochondrial autophagy axis

Autophagy plays a dual role in SASP regulation: while spatial coupling of autophagic catabolism and anabolism is essential for SASP maintenance via rapid protein turnover, its role in aging remains contentious (76). Autophagy and mitophagy appear to play context-dependent roles in aging and cancer, acting as adaptive quality-control mechanisms in some settings while contributing to therapy-associated stress responses and senescence maintenance in others. Notably, autophagy is critical for selective mitochondrial clearance (mitophagy), which prevents immune activation by removing dysfunctional yet structurally intact mitochondria. Age-dependent declines in mitophagy contribute to mitochondrial dysfunction, oxidative stress, and functional deterioration across tissues—a hallmark of aging (77, 78). Thus, impaired autophagic degradation, particularly mitophagy, exacerbates oxidative damage and age-related cellular dysfunction.

### Targeting PD-L1 glycosylation and redox imbalance

The immune checkpoint PD-L1 is upregulated in lung tissues of naturally aged mice, driving immune cell exhaustion and propagating PD-L1 expression in non-senescent cells via JAK-STAT pathway activation and SASP amplification (79). Verdura et al. demonstrated that resveratrol directly inhibits glucose-dependent PD-L1 glycosylation by targeting α-glucosidase/α-mannosidase activity, thereby disrupting N-linked glycan modifications, promoting PD-L1 dimerization, and synergistically enhancing antitumor T cell responses (80). Given the immunostimulatory and anti-inflammatory properties of exogenous antioxidants (e.g., vitamin D, C, E), combinatorial approaches pairing these agents with ICIs are under active investigation. Preclinical studies reveal synergistic efficacy, likely mediated by antioxidant-mediated restoration of mitochondrial function and metabolic homeostasis in aged immune cells (81–83). Therefore, antioxidant-based approaches may represent one potentially actionable component of a broader strategy aimed at restoring immune fitness and improving immunotherapeutic responsiveness in older patients.

### Senescence-targeted strategies

Senolytics and senomorphic agents may represent another potential approach to improve immunotherapy in older patients. Senescent cells can reinforce an immunosuppressive TME through SASP-driven inflammation, stromal remodeling, and immune-checkpoint ligand expression. Notably, PD-L1+ senescent cells accumulate with age, and PD-1 blockade has been shown to enhance CD8^+^ T cell-dependent clearance of these cells in preclinical models (84). Therefore, selective elimination of senescent cells or suppression of detrimental SASP programs may help reduce age-associated inflammatory and immunosuppressive cues. However, because senescence can exert both tumor-suppressive and tumor-promoting effects depending on context, senolytic–ICI combinations require careful timing, patient selection, and prospective validation (85).

### Aging-associated gut microbiome heterogeneity and its implications for immunotherapy

The human microbiota, comprising symbiotic and pathogenic microorganisms, is estimated to exceed 38 trillion cells—surpassing the number of human somatic cells. This microbiome-host symbiosis critically regulates diverse biological processes, including immunity and metabolism (86). Age-associated remodeling of the gut microbiota disrupts the gut-immune axis and may impair immunotherapy efficacy (87, 88). However, aging-related microbial changes are heterogeneous rather than uniformly detrimental. Recent evidence identified an aging-enriched enterotype associated with favorable responses to immune checkpoint blockade, whereas frailty-related microbial configurations were linked to less healthy aging trajectories (89, 90). These observations suggest that microbiome heterogeneity may partly contribute to variable immunotherapy outcomes in older patients.

Antibiotic-driven dysbiosis impairs ICI responses in murine models, reducing cytokine secretion and crippling CpG-oligonucleotide therapy efficacy (86, 91). Conversely, fecal microbiota transplantation (FMT) restores ICI effectiveness in germ-free mice, and clinical metagenomics links *Akkermansia muciniphila* abundance to improved anti-PD-1 outcomes (92). In addition, the efficacy of anti-PD-1 immunotherapy was markedly different in melanoma patients with greater gut microbial diversity and relative abundance (93), and specific beneficial bacteria such as *B. adolescentis* may further enhance anti-PD-1 responses (94). Because geography and habitual diet are major determinants of gut microbiome composition, region- and diet-specific microbial configurations in older adults may provide a practical entry point for improving immunotherapy efficacy (95). Thus, microbiome-directed strategies in elderly populations may need to be personalized according to dietary background, frailty status, and geographic context rather than applied uniformly (96). These findings position the aging-associated microbiome as both a biomarker and modulator of immunotherapy response, with therapeutic potential for microbiome-targeted interventions.

### Beyond checkpoint blockade: reprogramming the aged TME

Overall, optimizing immunotherapy for elderly patients requires more than simply enhancing PD-1/PD-L1 blockade; rather, it necessitates restoring the immune fitness of the aging host. Given the age-dependent accumulation of PD-L1 senescent cells and the presence of systemic inflammaging, a comprehensive therapeutic strategy—including the improvement of metabolic and mitochondrial function, restoration of autophagic and mitophagic homeostasis, correction of redox imbalance, and modulation of the gut microbiota—may act synergistically with ICIs, thereby enhancing therapeutic efficacy in older patients.

## Conclusion and future perspectives

Cancer treatment in elderly patients remains a formidable challenge, necessitating deeper insights into immunosenescence to optimize clinical outcomes. The immune system operates as a central coordinator of organismal health, dynamically interacting with the nervous, endocrine, and metabolic systems. Age-related immune remodeling—spanning both innate and adaptive immunity—is predominantly deleterious, impairing antitumor surveillance and therapeutic responses. In this sense, immunosenescence is not only the fundamental reason for the decline in the efficacy of ICI in elderly patients, but also an important determinant of the changes in the toxicity profile. Importantly, age-associated resistance to cancer immunotherapy is unlikely to be explained by a single mechanism. Rather, it emerges from the convergence of defective antigen presentation, progressive T cell dysfunction, myeloid and regulatory-cell-driven suppression, stromal remodeling, and host metabolic decline. In this context, antioxidant and metabolic interventions should be viewed not as stand-alone solutions, but as components of a broader framework aimed at restoring immune fitness in older patients. Future research must elucidate these cross-system interactions to develop interventions that mitigate aging-associated immune decline while harnessing protective mechanisms for healthy longevity. Although many complexities in this field remain unresolved, accumulating evidence indicates that advances in PD-1/PD-L1 blockade and aging biology are jointly clarifying the mechanistic basis underlying differential responses to immunotherapy. These insights not only provide a foundation for the development of predictive biomarkers and the design of age-stratified, mechanism-informed clinical trials, but also offer a feasible framework for optimizing individualized treatment strategies for older patients.
